# 2708. Infectious Complications Among CD19 Chimeric Antigen Receptor T-cell Recipients

**DOI:** 10.1093/ofid/ofad500.2319

**Published:** 2023-11-27

**Authors:** Jonathan Huggins, Julia A Messina, Jennifer Saullo, Tessa Andermann, Melody Smith, Daniel Schrum, Erin Eberwein, Erin Kennedy, Krista Rowe-Nichols, Christopher Kelsey, Taewoong Choi, Matthew McKinney, Ahmed Galal, Yubin Kang

**Affiliations:** Duke University Hospital, Durham, North Carolina; Duke University, Apex, North Carolina; Duke University Hospital, Durham, North Carolina; University of North Carolina, Chapel Hill, North Carolina; Stanford University, Stanford, California; Duke University Hospital, Durham, North Carolina; Duke University Hospital, Durham, North Carolina; Duke University Hospital, Durham, North Carolina; Duke University School of Medicine, Durham, North Carolina; Duke University Hospital, Durham, North Carolina; Duke University Hospital, Durham, North Carolina; Duke University Hospital, Durham, North Carolina; Duke University Hospital, Durham, North Carolina; Duke University Hospital, Durham, North Carolina

## Abstract

**Background:**

Current data on infectious complications of chimeric antigen receptor (CAR) T-cell therapy are limited to single center retrospective cohort studies. Presented here is a subset of patients from a multicenter retrospective cohort study including recipients of CAR T-cell therapy at Duke University, University of North Carolina at Chapel Hill, and Stanford University.

**Methods:**

In a retrospective cohort of 66 patients who received CD19 CAR T-cell therapy Duke University between January 1, 2018 and August 31, 2021 rates and characteristics of bacterial, viral, and fungal infections within the first year after CAR T-cell infusion are described. Demographic, baseline clinical, and outcome variables are compared between patients who developed infection and those who did not.

**Results:**

Forty-nine total infections occurred in 29 (43.9%) patients within 1 year of CAR T-cell infusion. Patients who developed infection were more likely to have an underlying malignancy other than diffuse large B-cell lymphoma (34% vs 8%, p = 0.031), and to have developed immune effector cell-associated neurotoxicity syndrome (62% vs. 32%, p = 0.016). Viral (23/49, 46.9%) and bacterial (20/49, 40.8%) pathogens predominated and infections most often involved the bloodstream (19/49, 38.8%) and lung (18/49, 36.7%).There were no statistically significant differences between rates of bacterial and viral infection based on time since CAR T-cell infusion (≤30 days vs 30 – 90 days vs >90 days), though fungal infections only occurred after 90 days in patients with relapse of their underlying disease.

**Figure:**
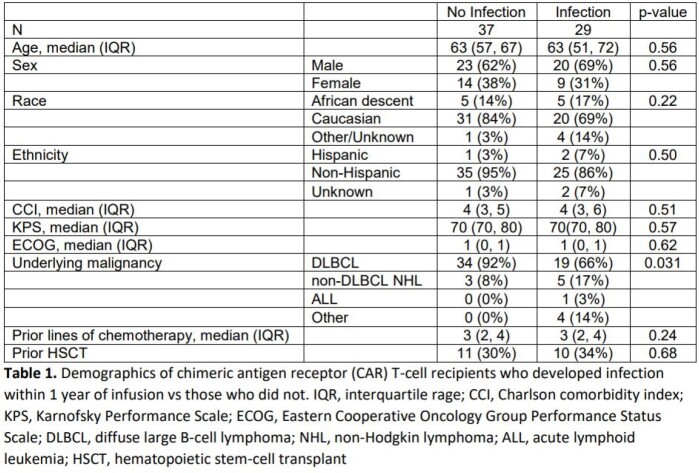

**Figure:**
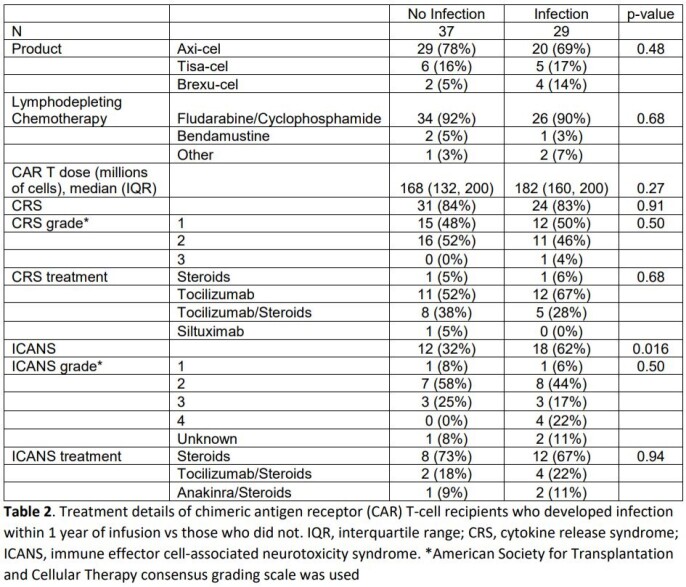

**Figure:**
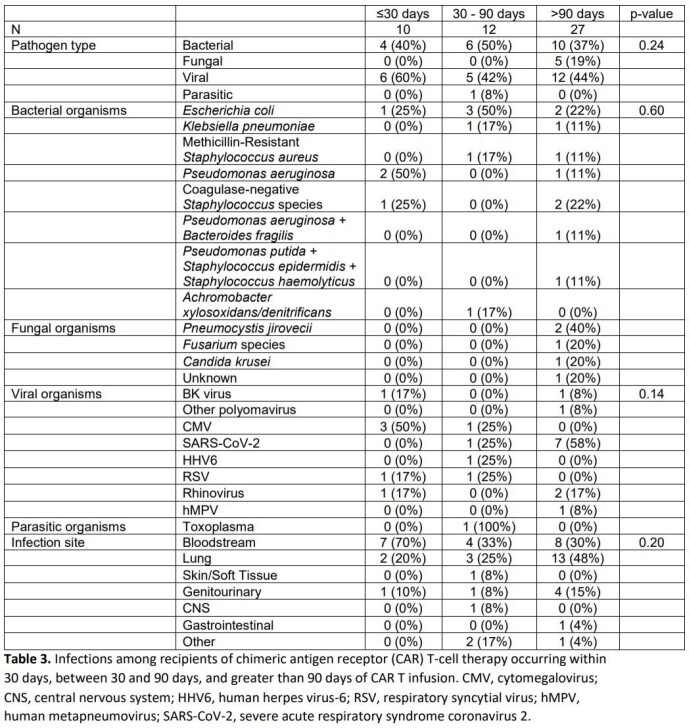

**Conclusion:**

Infectious complications after CAR T-cell therapy are common within the first year of infusion. Bacterial and viral infections predominate while fungal infections are rare and occurred only in patients with relapsed disease.

**Disclosures:**

**Melody Smith, MD, MS**, BMS: Advisor/Consultant **Krista Rowe-Nichols, RN, MSN, AOCNS**, Bristol Myers Squibb: Advisor/Consultant|Kite Pharmaceuticals: Advisor/Consultant|Kite Pharmaceuticals: Speaker Bureau **Christopher Kelsey, MD**, Colgate-Palmolive: Expert Testimony|Johnson and Johnson: Expert Testimony **Taewoong Choi, MD**, Janssen biotech: Honoraria **Matthew McKinney, MD**, ADC Therapeutics: Advisor/Consultant|ADC Therapeutics: Honoraria|Beigene: Grant/Research Support|Beigene: Honoraria|Epizyme: Advisor/Consultant|Genentech: Advisor/Consultant|Genentech: Grant/Research Support|Genentech: Honoraria|Gilead/Kite: Advisor/Consultant|Gilead/Kite: Honoraria|Incyte: Grant/Research Support|Novartis: Advisor/Consultant|Seagen, Inc.: Advisor/Consultant|Takeda: Advisor/Consultant|TG therapeutics: Advisor/Consultant

